# A Rare Case of Complex Hernia Causing Urethral Obstruction

**DOI:** 10.1055/s-0041-1736671

**Published:** 2021-12-15

**Authors:** Vivek Agrawal, Chinmay Bagla

**Affiliations:** 1 Department of Surgery, University College of Medical Sciences and Guru Teg Bahadur Hospital, New Delhi, India

**Keywords:** complex hernia, urethral obstruction, urethral deviation

## Abstract

Abdominal wall hernias rarely cause obstruction of the urinary tract. We present the case of a patient undergoing regular smooth dilatations for urethral stricture since 8 years who developed right inguinoscrotal swelling and narrowing of urinary stream since 2 years of age. There was a growing difficulty in dilatation due to path distortion of urethra by the hernia. He had a history of open suprapubic cystostomy (SPC) 8 years ago. The patient refused surgery till he landed with an inability to pass dilators and difficulty in passing urine. He was taken up for right inguinal exploration with internal optic urethrotomy (IOU). Intraoperatively, he was found to have right inguinal hernia with incisional hernia at the site of SPC which was repaired and a cystoscopy revealed urethral deviation with anterior urethral stricture for which IOU was done. Postoperatively, the patient's urethral tract straightened and his urinary complaints resolved. A complex hernia can cause urethral deviation and obstruction due to pressure effects of its contents and should be repaired at an early stage.

Abdominal wall hernias seldom cause obstruction of the urinary tract at the level of ureters and the bladder outlet. 1 2 We are reporting a case of a complex abdominal wall hernia, causing urethral path distortion leading to outflow obstruction in a patient having an old standing urethral stricture.

## Case Report

The patient was a 48-year-old male who had been undergoing regular dilatations for the last 8 years for sustaining relief from his urethral stricture. He had a history of having undergone open suprapubic cystostomy (SPC) 8 years ago for acute urinary retention secondary to recurrent urinary tract infection (UTI). He developed surgical site infection (SSI) postoperatively which was managed conservatively. The stricture had been managed with internal optic urethrotomy (IOU) and serial dilatations, following which the patient started doing well with 6 monthly dilatations.

Since last 2 years, he developed a swelling in the right inguinoscrotal region associated with repeated thinning of urinary stream and recurrent episodes of burning micturition. The swelling became static and persistent in the last 2 months.

On examination, there was a 5-cm long vertical midline scar in the suprapubic region. An irreducible soft swelling of 8 cm × 6 cm was present in the right inguinal region which extended up to the base of scrotum. Cough impulse was present, and we could not get above the swelling. The defect could not be localized. There was also a gradual growing difficulty in negotiating urethral dilators due to the path distortion by the contents of the hernia. Cystoscopy revealed narrowing in bulbar urethra with deviations in the proximal anterior urethra and the scope needed to be negotiated to reach the urinary bladder. Ultrasound showed findings consistent with right inguinal omentocele and a scarred suprapubic region. Defect could not be localized.

The patient had been coaxed for surgery multiple times but he did not agree due to poor past surgical experience. He eventually landed with difficulty in micturition and inability to get dilators passed. A preoperative diagnosis of right sided, complete, irreducible, inguinal omentocele with urethral stricture was made, and the patient was planned for right open hernioplasty with cystourethroscopy along with IOU.

**Fig. 1:**
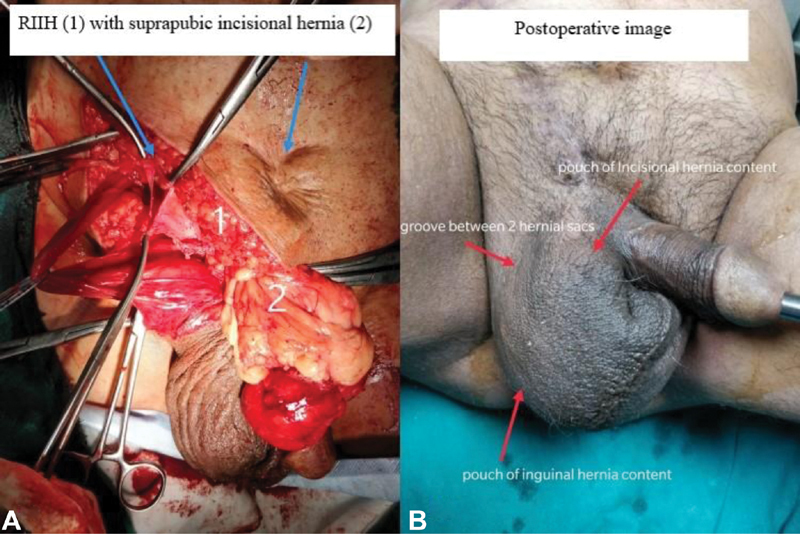
( **A** ) Intraoperative image showing the right indirect inguinal hernial sac and the contents of the suprapubic incisional hernia. ( **B** ) Postoperative image showing the visible surface markings of pouches of inguinal and incisional hernial sacs.

Intraoperatively, a right indirect hernial sac with omentum as content was found; the sac was opened, omentum resected, and sac was transfixed at its neck followed by posterior wall strengthening. Another hernial sac with omentum as content was found in midline beneath the scar of old SPC which was densely adherent to the surrounding fibrotic tissue ( Fig. 1A ). This sac was dissected out, opened, content resected, and sac was closed. For the right inguinal hernia, a mesh hernioplasty was done. After the closure of the external oblique aponeurosis, anatomical repair of midline defect was achieved and strengthening was done with overlay mesh covering the midline defect. On cystourethroscopy, a stricture was present from posterior part of penile urethra to bulbar urethra. IOU was done and 18-Fr Foley's catheter left in situ.

A postoperative diagnosis of right-sided, complete, irreducible, indirect inguinal hernia, and incisional hernia with omentum as contents with urethral stricture was made. The patient developed SSI on post operative day (POD)-2 which was managed by wound irrigation and oral antibiotics.

At follow-up after 1 month, a repeat cystourethroscopy was performed which showed fibrosis and narrowing at the site of IOU. A repeat IOU was done. The anterior urethra had straightened out and dilatation was simple and smooth. The patient now has a good urinary stream, no complaint of restriction in passage and dilatations have become a smooth affair. The patient has been on 4 monthly dilatation for the past year which have all been smooth and has now been called after 6 months for next follow-up.

## Discussion

This was a case of an inguinal, as well as incisional, hernia secondary to lower urinary tract obstruction, the contents of which led to further penile urethral passage distortion due to external compression. This led to repeated difficult traumatic dilatations till the dilators could no longer be passed.

Inguinal and incisional hernias have been known to cause obstruction to the urinary tract. Reported causes have been extrinsic compression of ureters by the hernial contents, involvement of ureters in the hernial sac, or the presence of the urinary bladder, or its diverticula as contents of the hernial sac causing bladder outlet obstruction. 1 2 3 The present case highlights the importance of lower urinary tract obstruction as a cause for hernia and also points toward the deviation and further obstruction of urethra due to the direct mass effect of the omentum lying in the hernial sacs. Such an effect seems to have occurred by descent of the hernial contents under the Colles' fascia.

The deviation of the urethra was accompanied by lower urinary tract symptoms. Dilatations were attempted to relieve the patient's urinary symptoms. The patient's initial unwillingness for surgery probably led to the formation of a complex hernia secondary to the stricture which might have been prevented, had the patient undergone a timely urethroplasty. The patient's further unwillingness for a hernia repair caused increasing difficulty in urethral dilatations. While urethral dilatations can cause urethral trauma and recurrent strictures, 4 the appearance of lower urinary tract symptoms (LUTS) with hernia, the patient's previous 6 years of uneventful dilatations, and cystoscopy showing urethral deviation indicate that the path distortion was responsible for repeated trauma during dilatations that led to an increase in the severity of stricture and was managed by IOU. An earlier hernia repair before significant path deviation of urethra could have prevented the need for repeat IOU, while a further delay could have led to obstructive uropathy, the need for an emergency SPC, and a need of future urethroplasty to manage the stricture.

This case also highlights the impact of surgeries and their postoperative events on the psychology of patients impeding patient compliance, leading to a higher morbidity associated with a complicated illness and its complex management.

## Conclusion

Lower urinary tract obstruction may lead to hernias and all hernia patients must be evaluated for the same. In complex hernias, to prevent urethral deviation and obstruction by external pressure of hernial contents which can cause added complications, an early hernia repair should be undertaken to provide relief.
